# Structural and dynamic insights into the energetics of activation loop rearrangement in FGFR1 kinase

**DOI:** 10.1038/ncomms8877

**Published:** 2015-07-23

**Authors:** Tobias Klein, Navratna Vajpai, Jonathan J. Phillips, Gareth Davies, Geoffrey A. Holdgate, Chris Phillips, Julie A. Tucker, Richard A. Norman, Andrew D. Scott, Daniel R. Higazi, David Lowe, Gary S. Thompson, Alexander L. Breeze

**Affiliations:** 1 Discovery Sciences, AstraZeneca R&D, Alderley Park, Macclesfield SK10 4TG, UK; 2 MedImmune, Granta Park, Cambridge CB21 6GH, UK; 3 Astbury Centre for Structural Molecular Biology, Faculty of Biological Sciences, University of Leeds, Leeds LS2 9JT, UK

## Abstract

Protein tyrosine kinases differ widely in their propensity to undergo rearrangements of the N-terminal Asp–Phe–Gly (DFG) motif of the activation loop, with some, including FGFR1 kinase, appearing refractory to this so-called ‘DFG flip'. Recent inhibitor-bound structures have unexpectedly revealed FGFR1 for the first time in a ‘DFG-out' state. Here we use conformationally selective inhibitors as chemical probes for interrogation of the structural and dynamic features that appear to govern the DFG flip in FGFR1. Our detailed structural and biophysical insights identify contributions from altered dynamics in distal elements, including the αH helix, towards the outstanding stability of the DFG-out complex with the inhibitor ponatinib. We conclude that the αC-β4 loop and ‘molecular brake' regions together impose a high energy barrier for this conformational rearrangement, and that this may have significance for maintaining autoinhibition in the non-phosphorylated basal state of FGFR1.

## Abstract (web summary)

Receptor tyrosine kinases are key mediators of cell proliferation that have been implicated in several disease states for which they represent promising drug targets. Here the authors determine the thermodynamic basis for the low propensity of FGFR1 to access the DFG-Phe-out conformation required to bind type-II inhibitors.

## Graphical abstract

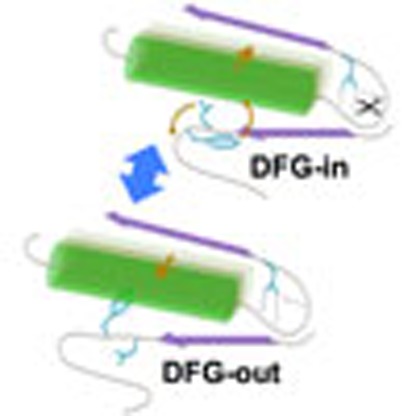

Receptor tyrosine kinases (RTKs) wield exquisite control over cell differentiation, fate, metabolism and homeostasis. Dysregulation of RTK signalling plays a significant role in the pathogenesis of disease conditions ranging from cancers to inflammatory and neurodegenerative illnesses. Hence, it is not surprising that over the past two decades they have become one of the most important classes of enzyme to be exploited as targets for drug discovery^1^. Conformational plasticity is an essential feature of kinase function and regulation. Inhibitors of kinase domain catalytic activity developed in the course of drug discovery programmes have drawn attention to the importance of mobility in the conserved Asp–Phe–Gly (DFG) tripeptide motif at the proximal end of the activation loop (A-loop). The majority of kinase inhibitors described to date bind competitively with ATP to a presumed basal state conformation (termed ‘DFG-in' or the ‘type I' binding mode) in which the Phe side chain of the DFG motif resides in a hydrophobic pocket deep within the kinase fold. An early insight into the role of the DFG motif as a conformational switch was provided by the structure of the tyrosine kinase Bcr-Abl complexed with the inhibitor STI-571 (imatinib)^2^. This structure indicated that the DFG motif undergoes a conformational rearrangement whereby the Phe side chain is flipped out of its hydrophobic pocket, vacating space for insertion of an aromatic moiety of the inhibitor. ‘DFG-out' conformations have since been observed in many kinases, both inhibitor bound^3^ and, occasionally, in the unbound state^4^,^5^,^6^,^7^,^8^. The DFG-out state is catalytically inactive, since it is sterically incompatible with cofactor and substrate binding, and in some kinases may natively contribute to autoinhibition^8^,^9^. Indeed, several so-called ‘type II' inhibitors, that bind to and stabilize the DFG-out form of a number of kinases, have been described^10^. An intriguing anecdotal observation from drug discovery is that it is relatively easy to identify type II (as opposed to type I) inhibitors against some kinases, but difficult or impossible for others. A plausible explanation for these differences may lie in the chemical space populated by screening libraries, favouring type I binding modes against some kinases and type II inhibitors in others. Alternatively, there could be specific structural or dynamic differences between individual kinases that relatively favour one or other binding mode. This conformational balance has been referred to as the ‘DFG-out propensity'^11^. Evidence has been advanced recently that DFG-out propensity and/or the rates of interconversion between DFG-in and DFG-out may be influenced by the side chain properties at, or adjacent to, particular points of the regulatory or catalytic ‘spines' of the kinase domain^12^.

Members of the FGFR family (FGFR1 to 4) are key mediators of both developmental and disease-associated angiogenesis^13^ and are heavily implicated in the pathogenesis of tumour vascularization in a number of different tumour types including breast^14^, pancreatic^15^, prostate^16^ and ovarian^17^ carcinomas, as well as being driving oncogenes for malignant transformation in their own right^13^,^18^. Hence, they have been seen as attractive targets for the development of therapeutic agents aimed at inhibition of tumour growth and metastasis. Despite concerted efforts to develop type II inhibitors of FGFR1 kinase in our own drug discovery programme, we obtained only type I inhibitors as confirmed by X-ray crystallographic analysis of >70 compounds, and none of the >30 FGFR1 kinase structures in the Protein Data Bank adopts the DFG-out conformation. Recently, however, we observed that the Bcr-Abl inhibitor ponatinib (AP24534) also binds potently to FGFR1 kinase, and moreover we and others have now confirmed that it binds to the DFG-out conformation of FGFR kinases^19^,^20^,^21^. Intrigued by this finding, we embarked on an investigation of the factors that underlie the seemingly strong preference for the DFG-in state in FGFR1, using inhibitors that stabilize the respective A-loop conformations as chemical ‘free-energy probes'. When compared with well-known type I inhibitors, binding of ponatinib to FGFR1 revealed startling differences in kinetic and thermodynamic behaviour associated with the two binding modes. Our analysis of changes in protein dynamics between the unbound, type I-bound and type II-bound states, using both nuclear magnetic resonance (NMR) and hydrogen–deuterium-exchange mass spectrometry (HDX-MS), shows that both proximal and distal structural elements influence activation loop conformational energetics in FGFR1.

## Results

**Figure 1:**
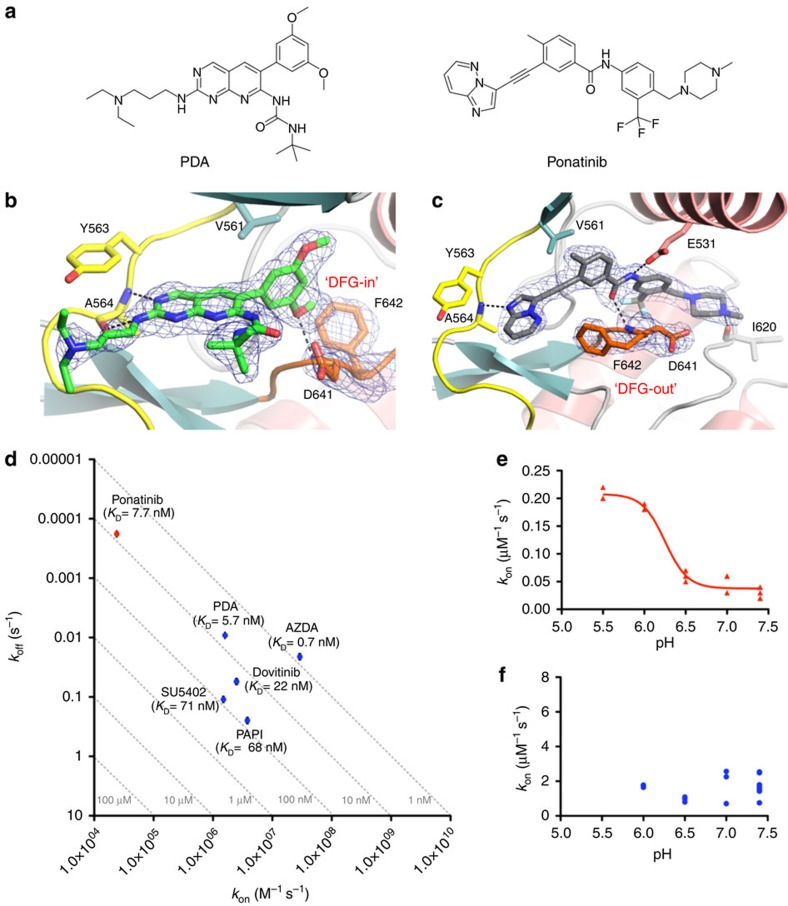
Structural and kinetic characteristics of FGFR1 complexes with the type I and type II inhibitors PDA and ponatinib. (**a**) Chemical structures of PDA and ponatinib. (**b**) Active site of FGFR1 kinase in complex with PDA (green carbons) as determined at 2.09 Å resolution (Supplementary Table 5). The hinge region (yellow) and A-loop (orange) are highlighted. *F*_o_−*F*_c_ OMIT electron density for PDA and the DFG motif is represented as a blue mesh contoured at 3.0*σ*. Polar interactions are indicated as dotted lines. (**c**) Active site of FGFR1 kinase in complex with ponatinib (grey carbons) as determined at 2.33 Å resolution, with *F*_o_−*F*_c_ OMIT electron density for ponatinib and the DFG motif represented as a blue mesh contoured at 3.0*σ*. Colouring of FGFR1 as in **b**. (**d**) Kinetic value plot of association rate constant (*k*_on_) versus dissociation rate constant (*k*_off_). Rate constants were determined using SPR at 298 K, pH=7.4. The affinities (*K*_D_) were calculated from the equation *K*_D_=*k*_off_/*k*_on_ and broken lines represent affinity isotherms. Data represent geometric means from at least three independent experiments; standard errors are shown as error bars (values and errors are presented in Supplementary Table 1). (**e**) The association rate constant of ponatinib binding to FGFR1 as a function of pH, as measured by SPR at 298 K. The red line represents the result of the non-linear fitting of the data to the 4 PL model (*R*^2^=0.968; p*K*_a_(Asp641)=6.25). (**f**) The association rate constant of PDA binding to FGFR1 as a function of pH, as measured by SPR at 298 K.

For our studies of the FGFR1 kinase domain, we have used a construct spanning residues Ala458 to Glu765 of human FGFR1 that contains two mutations (Cys488Ala and Cys584Ser) designed to stabilize the enzyme against covalent aggregation. The protein is non-phosphorylated after co-expression with PTP1B and purification from *Escherichia coli*. An additional mutation of the catalytic aspartate (Asp623Ala) was introduced for NMR studies to increase further the yield of stable isotope-labelled protein. Our previous studies have shown that this mutation does not detectably perturb the structure of the FGFR1 kinase domain^22^. In addition, we have confirmed using surface plasmon resonance (SPR) that binding parameters for a close analogue of the canonical FGFR1 inhibitor PD173074 (ref. 23; henceforth referred to as PDA; Fig. 1a; Supplementary Fig. 1) are unaltered for the Asp623Ala mutant relative to the kinase-active form (data not shown).

### Ponatinib-binding kinetics suggest a low DFG-out propensity

On binding of ponatinib (Fig. 1a), and in contrast to the binding of PDA, FGFR1 kinase domain was observed to adopt a DFG-out conformation as determined by X-ray crystallography in our laboratory and reported elsewhere^19^,^20^,^21^ (Fig. 1b,c). The reason for the apparently refractory behaviour of FGFR1 towards adopting ‘DFG-out' has, however, remained elusive to date. We hypothesized that an intrinsically low DFG-out propensity might be the underlying reason, prompting us to compare the binding kinetics of ponatinib (to date the only known high-affinity type II FGFR1 inhibitor) with those of representative type I inhibitors (Supplementary Fig. 1a). A kinetic analysis using SPR highlights the fact that while the type I inhibitors we investigated show fairly uniformly fast association rate constants, the binding of ponatinib is exceptionally slow (Fig. 1d, Supplementary Table 1). With an association rate constant of 2.4 × 10^4^ M^−1^ s^−1^, it is ∼70 × slower than that of PDA (*k*_on_=1.6 × 10^6^ M^−1^ s^−1^), which has almost identical affinity to ponatinib. Notably, the comparable affinity of ponatinib (*K*_D_=7.9 nM) and PDA (*K*_D_=5.7 nM) for FGFR1 is a result of the outstandingly long lifetime of the ponatinib–FGFR1 complex: the dissociation rate constant for ponatinib (*k*_off_=1.9 × 10^−4^ s^−1^, corresponding to a half-life (*t*_1/2_) for the complex of ∼61 min) is ∼50 × slower than that for PDA (*k*_off_=9.2 × 10^−3^ s^−1^; *t*_1/2_=1.3 min). Slow rate constants have previously been reported for type II inhibitors binding to a number of kinases^24^,^25^,^26^,^27^,^28^. These observations have been interpreted as consistent with a slow equilibrium between DFG-in and DFG-out states, where the DFG-out conformation is sampled only infrequently, accompanied in some cases by slow interconversion of ligand conformations, as ,for example, in the binding of analogues of BIRB-796 to p38 MAP kinase^29^. Recently, Shan *et al*.^11^, using Abl kinase as a model system, suggested that the DFG conformation is controlled by a protonation-dependent energetic switch. According to that analysis, the acidity of the DFG aspartate may be one factor that drives the equilibrium between the DFG-in and DFG-out conformations. To investigate whether protonation of the DFG aspartate also influences the DFG flip in FGFR1, we derived the association rate constants of the DFG-out inhibitor ponatinib and the DFG-in inhibitor PDA as a function of pH. The rate of ponatinib binding to FGFR1 increased nearly sevenfold as pH decreased from 7.4 to 5.5 (Fig. 1e). Ponatinib is expected to be protonated on its terminal methylpiperazinyl nitrogen across this pH range; thus, the pH dependence very likely reports on the ionization state of Asp641, giving a calculated effective p*K*_a_ of 6.25, well above the unperturbed range for aspartate. The observed variation in the on-rate for binding to the DFG-out conformation as a function of Asp641 ionization state lends support to the hypothesis that the DFG flip is rate-limiting on ponatinib association to FGFR1. In contrast to ponatinib, the binding kinetics of PDA to FGFR1 showed no dependence on pH over a similar range (Fig. 1f), which is consistent with the assumption that the binding of the so-called DFG-in inhibitors is not affected by the conformation of the DFG motif^30^.

### Ponatinib binding is accompanied by an enthalpic penalty

**Figure 2:**
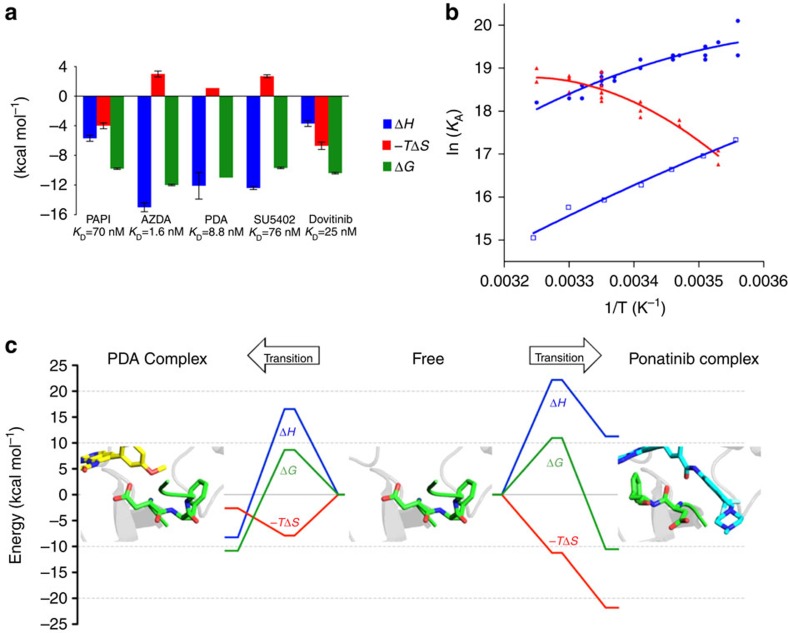
Thermodynamic data for inhibitors binding to FGFR1 kinase domain. (**a**) Thermodynamic signatures for type I inhibitors binding to FGFR1 derived by ITC at 298 K. Data shown are arithmetic mean±s.d. from at least two independent experiments (values and errors are presented in Supplementary Table 2). (**b**) van't Hoff plot visualization of temperature-dependent FGFR1–ligand interactions measured by SPR for PDA (blue circles), SU5402 (blue open squares) and ponatinib (red triangles). (**c**) Thermodynamic reaction pathway models for FGFR1 interacting with PDA (left) and ponatinib (right). The reaction coordinate depicts the lowest energy continuous pathway between the free (centre of the figure) and bound states (left for PDA complex; right for ponatinib complex) via the transition state, for free energy Δ*G* (green), enthalpy Δ*H* (blue) and entropy—*T*Δ*S* (red).

The apparently slow equilibrium between DFG-in and DFG-out conformations in FGFR1 kinase suggests a high free-energy barrier for the DFG flip. We carried out a detailed analysis of the changes in enthalpy and entropy that accompany ligand binding to enhance our understanding. For the selected type I inhibitors, isothermal titration calorimetry (ITC) experiments revealed exothermic binding reactions (Fig. 2a, Supplementary Fig. 2, Supplementary Table 2), and the derived binding affinities were largely in agreement with those determined by SPR. In contrast, for ponatinib, which binds to FGFR1 in a DFG-out conformation, the observed titration curve (Supplementary Fig. 2h) was of poor quality and did not allow derivation of thermodynamic parameters. As an alternative to ITC, we analysed kinetic and equilibrium data from SPR as a function of temperature, following the van't Hoff method, to provide independent thermodynamic characterization of binding events. For ponatinib and two selected type I inhibitors (PDA and SU5402), the derived binding enthalpies and entropies revealed another marked difference between the type II inhibitor ponatinib and the selected type I inhibitors (Fig. 2b, Table 1). In the case of PDA and SU5402, van't Hoff analysis confirmed exothermic binding enthalpies (PDA, Δ*H*=−11.5 kcal mol^−1^; SU5402, Δ*H*=−14.2 kcal mol^−1^) and the data are in close agreement with the Δ*H* values of −12.1 kcal mol^−1^ (PDA) and −12.4 kcal mol^−1^ (SU5402) determined by ITC. Unexpectedly, the type II inhibitor ponatinib showed an endothermic Δ*H* value (Δ*H*=10.1 kcal mol^−1^) that indicates enthalpically unfavourable binding. Ponatinib and PDA exhibit comparable van't Hoff free energies of binding (Table 1) that are consistent with their very similar affinities measured directly by SPR; however, breaking this down into enthalpic and entropic components revealed significant differences, as the binding of PDA and ponatinib were determined to be enthalpy driven and entropy driven, respectively. An endothermic enthalpy, as observed for the equilibrium between free and FGFR1-bound ponatinib, raises the possibility that the conformational rearrangement required to effect the DFG flip in FGFR1 may also be associated with an enthalpic penalty (neglecting net contributions from protein–ligand and protein–solvent interactions of ponatinib). Furthermore, we established that the vascular endothelial growth factor receptor (VEGFR) inhibitor tivozanib (AV-951) also binds to FGFR1 in a DFG-out mode (*K*_D_=1.3 μM by SPR) and does so endothermically by van't Hoff analysis (Supplementary Fig. 3), lending further support to the notion that this may be a signature of a DFG-out binding mode for FGFR1, rather than a compound-specific characteristic of ponatinib.

### A large free-energy barrier for the the DFG flip

By measuring the temperature dependence of the kinetic association and dissociation rate constants for PDA and ponatinib, we were able to discern the transition state energies for the association and dissociation steps of the binding reaction according to the method of Eyring (for details, see Supplementary Information). From linear Eyring plots (Supplementary Fig. 4), we determined Δ*H*^*#*^, −*T*Δ*S*^*#*^ and Δ*G*^*#*^ for the association and dissociation steps (Supplementary Table 3), which enabled us to construct detailed thermodynamic reaction pathway models for the binding of PDA and ponatinib to FGFR1 (Fig. 2c). For the binding of the type I inhibitor PDA to FGFR1, we observed a free-energy barrier of 8.7 kcal mol^−1^ associated with the transition state. This energy barrier is dominated by an enthalpic penalty (Δ*H*^#^_ass_=16.6 kcal mol^−1^); however, a significant favourable entropy (−*T*ΔS^#^_ass_=−7.9 kcal mol^−1^) lowers the overall free-energy barrier to reach the transition state. As observed for PDA, the transition state for binding of the type II inhibitor ponatinib to FGFR1 is also associated with an enthalpic penalty, which is partly compensated by a favourable entropic contribution. However, although the transition state entropy for the association of ponatinib (−*T*ΔS^#^_ass_=−11.2 kcal mol^−1^) is more favourable compared with that of PDA, it is not sufficient to compensate for the extraordinarily unfavourable Δ*H*^#^_ass_ of 22.2 kcal mol^−1^ for ponatinib binding, resulting in a 2.3 kcal mol^−1^ higher transition state free-energy barrier (Δ*G*^#^_ass_=11.0 kcal mol^−1^) associated with the type II binding mode. This difference in transition state free energy is in excellent accord with the ∼70-fold slower association rate constant that we measure for ponatinib binding.

### A partially unfolded intermediate in the DFG-out transition

Localized unfolding is widely believed to contribute to the crossing of free-energy barriers encountered during protein motion^31^, and ‘cracking' at the kinase hinge region has been observed to be a key element in the simulated DFG-in/out transition of EGFR kinase^32^. Assuming that protein conformational energetics contribute substantially to the free-energy changes on binding type I and type II inhibitors^33^, the thermodynamic signature (unfavourable enthalpy and favourable entropy) that we have observed for the association of ponatinib to FGFR1 (Fig. 2, Table 1) is consistent with the proposed model of partial unfolding, facilitating conformational transitions in proteins. Therefore, our thermodynamic data suggest that the transition state conformations traversed by FGFR1 during the ‘in-out' trajectory may be partially, or locally, unfolded. The guanidinium chloride (GdmCl)-induced unfolding transition curve of FGFR1 monitored by the change in far-ultraviolet circular dichroism (CD) shows two folding transitions, the first occurring between 1 and 2 M GdmCl with accumulation of an intermediate at ∼2 M GdmCl. The second transition occurs between 2 and 3.5 M GdmCl, by which point the protein is completely unfolded (Supplementary Fig. 5). Using SPR, we determined the association rate constant of ponatinib in the presence of 1.2 M GdmCl in the phosphate-buffered saline (PBS) running buffer to sample the partly unfolded intermediate. With an association rate constant of 2.3 × 10^5^ M^−1^ s^−1^, it is almost an order of magnitude faster than that of ponatinib in the absence of GdmCl (*k*_on_=2.4 × 10^4^ M^−1^ s^−1^). In contrast, PDA binding is minimally affected by the presence of 1.2 M GdmCl, with a *k*_on_=6.8 × 10^5^ M^−1^ s^−1^, consistent with the lack of requirement for a flip of the DFG motif for type I inhibitor binding. The structural loosening induced by intermediate concentrations of denaturant might be expected also to influence dissociation rates, and this was indeed observed for both inhibitors. Interestingly, the *k*_off_ for PDA was again only moderately affected (*k*_off_=1.8 × 10^−3^ s^−1^, against 9.2 × 10^−3^ s^−1^ in the absence of GdmCl; resulting in a *K*_D_ ∼2-fold weaker), while that for ponatinib was dramatically increased (*k*_off_=1.8 × 10^−2^ s^−1^ against 1.9 × 10^−4^ s^−1^ in the absence of GdmCl), leading to an ∼10-fold weaker *K*_D_ overall in the presence of 1.2 M GdmCl. The differential effects on association rate constant for DFG-in and DFG-out ligands provide evidence that the partly unfolded FGFR1 intermediate observed from the unfolding curve favours attainment of the DFG-out conformation, and furthermore suggests that it could serve as an intermediate of the DFG flip in FGFR1.

### Ponatinib binds to FGFR1 more slowly than to Abl

Ponatinib binds to DFG-out conformations of FGFR1 and Abl kinases with an almost identical binding mode and many conserved interactions between inhibitor and protein. In view of these overall similarities, we compared the ponatinib association rates for both kinases, using identical SPR-based protocols, to address the question of whether different underlying DFG-out propensities might play a significant role in type II inhibitor binding in these tyrosine kinases. The binding rate of ponatinib using SPR is over an order of magnitude faster for Abl (*k*_on_=5.2 × 10^5^ M^−1^ s^−1^) than for FGFR1 (*k*_on_=2.4 × 10^4^ M^−1^ s^−1^). This faster association rate accounts for the greater part of the roughly 10-fold higher affinity of ponatinib for Abl (*K*_D_=0.9 nM) than for FGFR1 (*K*_D_=7.9 nM), as the dissociation rate constants are rather similar for the two kinases (Supplementary Table 4). Our measured association rate for binding of the canonical type II inhibitor, imatinib, to Abl (*k*_on_=5.5 × 10^5^ M^−1^ s^−1^) is similar to that for ponatinib, and is in good accord with previously reported data^34^. This suggests that the difference in on-rate constant between the two kinases that we observe for ponatinib may reflect a fundamental difference in conformational energetic balance, with a considerably higher free-energy barrier for adopting the DFG-out conformation in FGFR1 in contrast to Abl kinase.

### Dynamic cross-talk revealed by NMR and mass spectrometry

**Figure 3:**
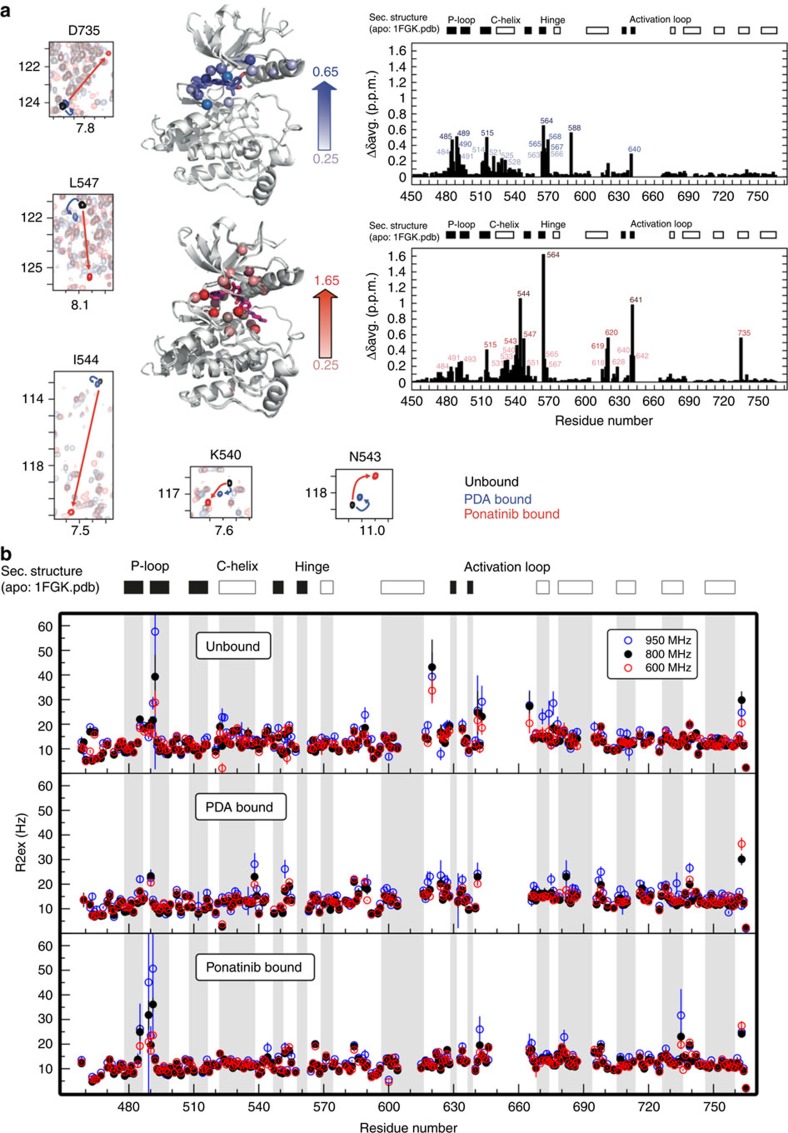
NMR analysis of structural and dynamic perturbations to FGFR1 kinase on binding of type I and type II inhibitors. (**a**) Backbone amide chemical shift perturbation (CSP) analysis on ligand binding. Weighted CSPs were calculated as Δ*δ*_ave_=(Δ*δ*^2^(N)/50+Δ*δ*^2^(H)/2)^1/2^ between unbound and PDA complex (top right panel), and between unbound and ponatinib complex (bottom right panel). The CSPs >0.25 for the two complexes are mapped on the X-ray crystal structure of unbound FGFR1 (PDB-code: 1FGK). Solid bars represent regions of β-strand secondary structure, open bars regions of α-helical secondary structure. Selected regions of overlayed ^1^H-^15^N TROSY-HSQC plots of representative amino acids in the αC-β4 loop and D735 in the distal αH helix are shown in small panels (left, bottom). The contour plots are colour coded as follows: unbound (black); PDA bound (blue); ponatinib bound (red). Arrows of the corresponding color connect the same residue in different spectra. (**b**) Analysis of chemical exchange contributions to transverse relaxation rates (*R*_2,ex_) measured for ligand-free (top), PDA-bound (middle) and ponatinib-bound (bottom) FGFR1 kinases at static fields of 600 MHz (black), 800 MHz (red) and 950 MHz (blue circles), reflecting motions on time scales >100 μs.

To gain insights into the dynamic origins of slow access to the DFG-out state in FGFR1, we employed both NMR spectroscopy and HDX-MS. We have previously reported NMR resonance assignments for FGFR1 kinase domain in the ligand-free state^22^. Titration of either PDA or ponatinib into samples of ^15^N-labelled FGFR1 kinase resulted in amide chemical shift perturbations (CSPs) in the slow-exchange regime that were completely saturated at 1:1 molar stoichiometry, typical of high-affinity binding in the nanomolar *K*_D_ range (Fig. 3a). Unlike for the unbound^22^ and the PDA-complex states of FGFR1, the first six residues (Asp641–Arg646) of the A-loop were observable in the ^1^H-^15^N TROSY-HSQC spectrum of the FGFR1–ponatinib complex, indicative of altered A-loop dynamics in the ponatinib complex compared with the unbound or PDA-bound kinase. Comparison of ^1^H-^15^N TROSY-HSQC spectra of both PDA and ponatinib complexes with unbound FGFR1 showed large amide chemical shift changes for many residues. Mapping of these perturbations onto the crystal structure of FGFR1 (PDB-code: 1FGK) shows that most of them are localized in the catalytically important and structurally conserved regions surrounding the active site (Fig. 3a). Significant CSPs were observed for Ala564 in the hinge region of both complexes, due to direct hydrogen-bond interactions with a ring nitrogen of the inhibitor; that seen in the ponatinib complex is substantially larger and may reflect a stronger hydrogen bond. For the PDA complex, CSPs were detected only for residues in the region of the P-loop, the N-terminus of the αC helix, the hinge region residues, and Ala640, which are all in close promixity to the inhibitor (Fig. 3a, upper right panel). Interestingly, ponatinib binding revealed both local and distal changes (Fig. 3a, lower right panel). Local CSPs were observed in the P-loop, αC helix and hinge regions, and for Ile620 in the catalytic loop all of which participate in direct interactions with the inhibitor. The backbone amide nitrogen of Asp641 (of the DFG motif) also engages in a direct hydrogen-bond interaction with the amide carbonyl oxygen of ponatinib, which is likely to dominate the observed CSP for this residue, along with the change in the φ torsion angle associated with the DFG flip (Fig. 3a, lower right panel; Fig. 1c). Notably, substantial CSPs were also observed in the αC-β4 loop around Ile544, and for Asp735 in the αH helix. These are all spatially distant from the active site; thus, the observed chemical shift changes (Fig. 3a, small panels) must be a result of structural or dynamic changes propagated through an interaction network. The CSPs in the αC-β4 loop region are of particular interest, since these amides are likely to be highly sensitive reporters on changes in conformation or dynamics associated with movements of the αC helix^35^. By analogy with other kinases, the hydrophobic spine network^36^,^37^,^38^ of FGFR1 is expected to be disrupted on the reorientation of Phe642 in the inactive DFG-out state, which may be reflected in perturbations seen in the chemical shifts of the residues neighbouring His621 in the catalytic loop. Direct contacts with the terminal methylpiperazinyl group of ponatinib from residues including Ile620 and His621 are also likely to contribute to the observed CSPs. Such perturbations are not seen for the PDA-bound state (which is assumed to populate predominantly the DFG-in conformation in solution). The large chemical shift change we observe for Asp735 in the ponatinib-bound complex is surprising, as Asp735 is situated in helix αH, which is rather remote from the active site. The upfield shift of the backbone amide resonance might reflect subtly altered hydrogen bonding and may report on perturbed dynamics in the αH helix as opposed to gross conformational change (*vide infra*), since the mean structures from X-ray crystallography are essentially superimposable in this region. Further insights into the underlying dynamics of FGFR1 in the three states were obtained from measurements of contributions from chemical exchange effects to the ^15^N transverse relaxation rates of backbone amides, *R*_2,ex_. Using data acquired at three different magnetic field strengths for unbound, PDA-bound and ponatinib-bound FGFR1, we observe particularly large field-dependent chemical exchange contributions to the ^15^N linewidth (attributable to dynamics on time scales longer than ∼100 μs) for the ponatinib complex in the P-loop, compared with smaller but still significant effects for unbound FGFR1, and a marked suppression of millisecond time-scale P-loop dynamics in the PDA complex (Fig. 3b); this correlates with the additional P-loop protein–ligand contacts that we observe in crystal structures of PDA-bound FGFR1, but also suggests that DFG-out binding of ponatinib is accompanied by loosening of restraining forces on P-loop conformation. However, in contrast to the enhanced P-loop dynamics, slow time-scale motions are markedly suppressed in the αC-helix of the ponatinib complex compared with either ligand-free or PDA-bound states. The *R*_2,ex_ data further show the presence of significant slow time-scale motion in the αH helix region of the DFG-out ponatinib complex around Asp735, in agreement with CSP data.

**Figure 4:**
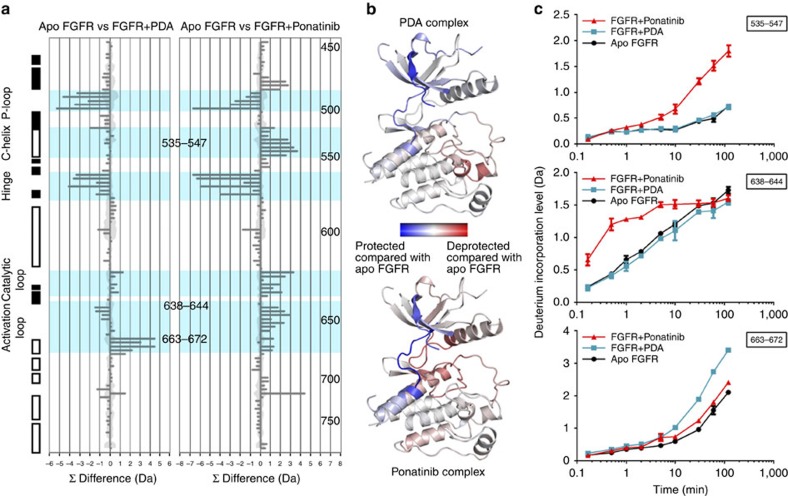
Hydrogen/deuterium-exchange changes on ligand binding to FGFR1 kinase domain. (**a**) Difference in hydrogen exchange relative to unbound FGFR1 for complex with PDA (left) and ponatinib (right). Protection due to ligand binding leads to a reduction in mass relative to the ligand-free form (more negative value); deprotection results in an increase in relative mass (more positive value). Each horizontal bar represents a single peptide from FGFR1. Vertical scale is not linear: peptides are in order of start residue from N (top) to C terminus (bottom). Peptides whose start residue is within a secondary structural element are indicated as filled (β-sheet) or empty (α-helix) bars. Values are the sum of all nine time points sampled (each is a minimum of two experiments and one to seven ions per peptide). Continuous shaded region denotes error at 1 s.d. Peptides from **c** are annotated by residue number. (**b**) Data from **a** as a heat map projected on the unbound FGFR1 structure (PDB-code: 1FGK): complex with PDA (top) and complex with ponatinib (bottom). Only significant changes are shown (>0.4 Da difference from ligand-free form per data point)^43^. Data sets have been normalized to the same scale. (**c**) Deuterium uptake plots for three peptides: residues Met535-Leu547, Lys638-Leu644 and Pro663-Leu672. Data points are the mean of at least two experiments. Error bars indicate 1 s.d.

Amide protection rates as measures of solvent accessibility determined by HDX-MS^39^,^40^ can provide complementary insights into conformational flexibility. By comparing the deuterium incorporation in the PDA and ponatinib complexes of FGFR1 with the unbound form (Fig. 4), several regions can be seen to exhibit significant relative (de)protection. Both inhibitor complexes are protected relative to ligand-free FGFR1 in the P-loop and inter-lobe hinge, consistent with direct protection from solvent by the ligand (Fig. 4b). The observed rate of hydrogen exchange in the P-loop follows the order unbound>ponatinib-bound>PDA-bound, reflecting the direct interaction between the *t*-butyl group of PDA and the P-loop. By comparison with the unbound and PDA-bound forms of FGFR1, the DFG-out ponatinib complex has faster exchange kinetics in the proximal A-loop, including the DFG motif (Fig. 4c middle panel). In contrast, the distal stretch of the A-loop, including the short αEF helical segment, displayes the opposite sensitivity to DFG-in or DFG-out binding modes: it is significantly deprotected in the PDA complex (Fig. 4c lower panel), whereas in the ponatinib complex this deprotection is marginal relative to ligand-free FGFR1. This finding may indicate a certain mutual exclusivity in the dynamic perturbations of the proximal and distal sections of the activation loop, which is not obvious from the X-ray crystal structure data in these regions.

Consistent with NMR chemical shift analysis, His621 in the catalytic loop and Phe642 (of the DFG motif) display increased hydrogen exchange in the ponatinib complex. Ponatinib-bound FGFR1 experiences widespread loss of hydrogen-exchange protection factors in peptides throughout the regulatory spine (R spine)^36^,^37^. Indeed, of the five amino acids in the R spine, four exhibit significant increases in observed hydrogen exchange rate in the ponatinib-bound ensemble (Fig. 4, Supplementary Fig. 9). In contrast, just one amino acid (His621) in the R spine was seen to have been marginally deprotected in the PDA-bound ensemble, while Phe642 was significantly protected. Together, these alterations to FGFR1 solvent accessibility indicate that the hydrophobic spine network is perturbed when the kinase adopts the DFG-out conformation. Significantly, and in agreement with NMR chemical shift data, another region that showed relative deprotection in the ponatinib-bound complex was the C-terminal end of the αC helix and the subsequent αC-β4 loop (Fig. 4c top panel). While the uncomplexed and PDA-bound forms show equivalent hydrogen-exchange profiles, the ponatinib complex displays a markedly greater extent of solvent exposure in this region on average. Again consistent with NMR, the αH helix also exhibits slight deprotection in the ponatinib complex, further supporting the likelihood of a structural loosening of this distal region of the kinase in the DFG-out state that is not evident from X-ray crystal structures.

## Discussion

The flip between active DFG-in and inactive DFG-out states of kinases, besides being exploitable for inhibitor design, has been advanced as a physiologically significant conformational transition that may have a role in modulation of the enzymatic activity of many kinases^11^. This is corroborated by the observation of DFG-out conformations in X-ray crystal structures of the unliganded and/or autoinhibited states of a number of kinases including Abl, c-Kit, FLT3, insulin receptor kinase and B-Raf^4^,^5^,^6^,^7^,^8^. The influence of the protonation state of the DFG aspartate on the *k*_on_ for binding of imatinib to the DFG-out state of Abl has been interpreted as a possible factor in the regulation of kinase activity through facilitation of nucleotide binding and release, and as evidence for a physiological role for the DFG flip^11^. Our results support a role for the protonation state of the DFG aspartate in influencing the accessibility of the DFG-out conformation in FGFR1, but the modest difference in p*K*_a_ of the DFG aspartate in FGFR1 (6.25) compared with that calculated for Abl (6.6)^11^ is insufficient to explain the wide gulf in *k*_on_ for type II binding to the two kinases. Our kinetic and thermodynamic data strongly suggest that, in contrast to Abl^41^, association of type II inhibitors is limited by an exceptionally slow DFG flip in FGFR1, because a particularly high free-energy barrier must be crossed in the transition between DFG-in and DFG-out states. Thus, there are likely to be structural and/or dynamic differences between Abl and FGFR1 that influence the accessibility of the DFG-out state. Evidence from Eyring analysis for the elevated free energy associated with the transition state is corroborated by the slow association kinetics for the type II inhibitor ponatinib, and by the differential effects on association and dissociation rate constants for ponatinib and PDA under conditions of partial unfolding or structural loosening in the presence of 1.2 M GdmCl. This calls into question whether it is feasible that such an innately slow DFG flip could play a physiologically relevant role in the catalytic function of FGFR1, as has been postulated for other kinases^4^,^5^,^6^,^7^,^8^.

The thermodynamic signatures for binding to the DFG-in and DFG-out states of FGFR1 appear to be highly distinct, with favourable enthalpy (at relative entropic cost) for PDA binding contrasting with a highly entropically driven interaction for ponatinib. This rather extreme example of enthalpy–entropy compensation^42^ between two inhibitors sharing very similar *K*_D_s but strikingly different binding modes may point to greater motional freedom as a contributory factor in the energetics of binding of ponatinib to the DFG-out state. Indeed, our HDX-MS data indicate an overall increased exposure of backbone amides to solvent in the DFG-out complex relative to unbound FGFR1, compared with predominantly enhanced protection from exchange in the DFG-in complex with PDA. This loosening of the structure in the DFG-out conformation is reflected in the region of the αH helix, where we observed a large NMR shift for Asp735, increased chemical exchange contributions to the NMR *R*_2_ relaxation rates and enhanced hydrogen exchange rates for surrounding residues in HDX-MS experiments, despite essentially identical mean conformations as judged by X-ray crystallography. We speculate that this effect is mediated through the αF helix, which anchors the hydrophobic spine network. Loss of communication through the spine as a result of the DFG flip may lead to slight destabilization of the αH helix, resulting in increased dynamic freedom in this region. We hypothesize that the increased mobility evident from hydrogen exchange and NMR relaxation data in regions both proximal (P-loop) and distal (αH helix) to the ponatinib-binding site and the DFG motif may contribute enhanced protein conformational entropy towards the markedly favourable gain in global entropy that characterizes the formation of the ponatinib complex^33^,^43^,^44^. Conversely, the suppression of slow time-scale motion in the αC helix that we see from NMR *R*_2_ relaxation rates in the ponatinib-bound state may contribute to the long residence time that characterizes the DFG-out binding mode.

**Figure 5:**
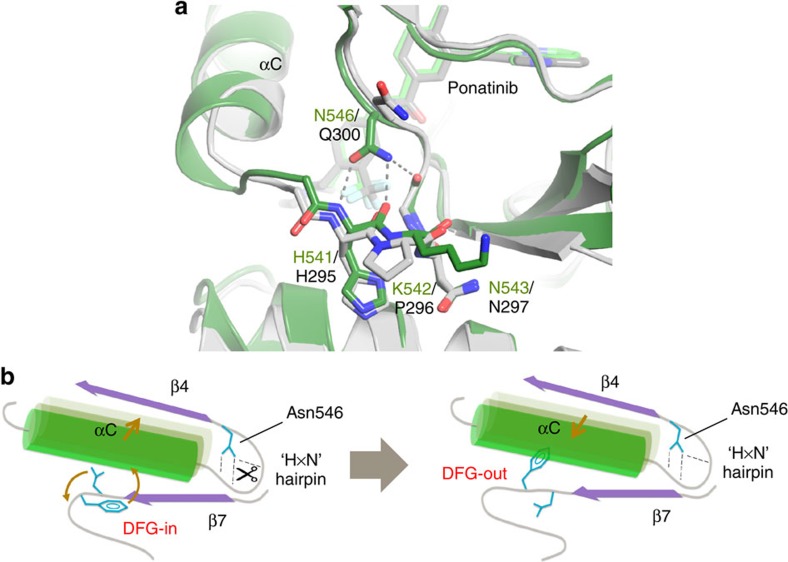
The role of the αC-β4 loop and molecular brake regions in the DFG flip of FGFR1. (**a**) Comparison of αC-β4 loop and molecular brake regions in FGFR1 and Abl kinase complexes with ponatinib. The structures of the FGFR1/ponatinib complex (PDB ID: 4V01) and the Abl/ponatinib complex (PDB ID: 3OXZ) are displayed in dark green and grey, respectively, with the bound ponatinib inhibitors displayed, respectively, in light green and grey. Relative to Abl, the αC helix of FGFR1 extends approximately one-half turn further, in part due to insertion of a Gly at position 539, and the ‘HxN hairpin' contains a Lys rather than a Pro at the middle position. The molecular brake of FGFR1 is engaged via hydrogen bonds (dotted lines) from the side chain of Asn546 and likely inhibits the outward motion of helix αC in FGFR1, whereas Abl, with a Gln at the equivalent position and lacking the Gly insert at position 539, is unable to form the molecular brake interactions. (**b**) Schematic illustration of the interplay between the DFG flip, outward movement of the αC helix and the proposed role of the Asn546 molecular brake hydrogen bonds in FGFR1. The Asn546 hydrogen bonds (of which Abl lacks an equivalent) may need to be transiently disengaged (scissors) to facilitate the αC-out, and hence DFG-out, movements. View in **b** as if from the left-hand side of **a**.

Crystal structures and molecular dynamics simulations, using Abl kinase as a model system, suggest that displacement of the αC helix away from the active site facilitates the DFG flip in kinases, with the resulting ‘αC-out' conformation being a potential intermediate^11^,^36^. The αC-β4 loop has been proposed to act as an anchor for the αC helix to the catalytic core, and as a hinge for the αC helix during the transition from active to inactive states of protein kinases^34^,^45^. The substantial amide CSPs we observed using NMR for residues in the αC-β4 loop in the DFG-out state of FGFR1, coupled with significantly enhanced solvent exchange rates by HDX-MS, indicate that the transition from the active to the inactive state is accompanied by a structural or dynamic perturbation. Compared with Abl, FGFR1 contains an insert (Gly539) C-terminal to the αC helix and a conformationally significant substitution in the relatively conserved ‘HxN hairpin'^35^,^46^ that follows (HPN in many kinases including Abl; HKN in FGFRs). The HxN hairpin may function as a pivot for the outward movement of the αC helix that is required to facilitate the excursion of the DFG Phe side chain towards its ‘out' configuration^46^. The Gly539 insert results in extension of the C-terminal end of the αC helix of FGFR1 by around half a turn relative to Abl (Fig. 5a), and facilitates the formation of the molecular brake hydrogen-bond network^47^ between the side chain of Asn546 and the backbone atoms of His541 of the HxN motif. By contrast, Abl is unable to form these hydrogen bonds to the HxN backbone. Asn546 is a key member of the triad that forms the molecular brake in FGFR isoforms, and is the site of a number of pathogenic gain-of-function mutations that are implicated in developmental disorders and cancers. The hydrogen–bond network involving Asn546 of FGFR1 would be expected to stabilize the αC helix in its ‘in' orientation, thereby inhibiting the 'αC-out' movement required to effect the DFG flip (Fig. 5b). Thus, our analysis suggests that a distributed network of individual contributions from several regions of the kinase structure conspires to hinder the DFG flip in FGFR1, and that the most important of these is likely to reside in the αC-β4 loop region. This is interesting in light of a recent report that the N550K mutation in FGFR2 (equivalent to Asn546 in FGFR1) confers resistance to the type I inhibitors PD173074 and dovitinib, but not to ponatinib, which displays enhanced inhibitory potency against this mutant relative to wild-type in BaF3 cell proliferation assays^48^. Our insights into the structural and dynamic influences on the DFG flip in FGFR1 corroborate the important role of the molecular brake in inhibiting basal kinase activity in unphosphorylated FGFRs, and imply that its function (and its release by pathogenic mutations) may be intimately associated with its ability to suppress the catalytically significant DFG flip^11^ by inhibiting the outward movement of the αC helix.

## Methods

### Protein expression and purification

Human FGFR1 consisting of residues Ala458-Glu765 with an engineered TEV-cleavable N-terminal 6 × His tag and mutations Cys488Ala and Cys584Ser was co-expressed in *Escherichia coli* with protein tyrosine phosphatase 1B (PTP1B) and purified by sequential immobilized metal affinity chromatography (IMAC, QIAGEN NiNTA), ion exchange (ResourceQ) and size exclusion chromatography^49^. The hexa-histidine tag was cleaved from protein by overnight treatment with TEV protease and concomitant dialysis, immediately after the IMAC step. Purified protein in a buffer comprising 20 mM Tris-HCl, pH 8.0, 20 mM NaCl, 2 mM TCEP, was snap frozen in liquid nitrogen and stored at −80 °C. For NMR studies, an additional mutation (Asp623Ala) was introduced to improve the yield of stable isotope-labelled FGFR1 kinase protein^22^. Uniform isotopic labelling was achieved by growing *E. coli* BL21 (DE3) Star cells in D_2_O-based M9 minimal medium supplemented with ^15^NH_4_Cl (Cambridge Isotope Laboratories or Sigma Aldrich) together with U-[^1^H,^13^C]- or (for fully deuterated samples) U-[^2^H,^13^C]-glucose (Cambridge Isotope Laboratories or Sigma Aldrich) as sole nitrogen and carbon sources, respectively. Purification was by IMAC and ion exchange chromatography; the 6 × His tag was not cleaved from the protein used for NMR. Human Abl consisting of residues Ser248-Val534 with an engineered TEV-cleavable N-terminal 6 × His tag and mutation Asn355Ser was expressed and purified as described^50^ with minor modifications. For biophysical studies, the 6 × His tag was retained intact for both kinases as it was used for immobilization on a nitrilotriacetic acid (NTA) sensor chip.

### Crystallization, crystallographic data collection, structure determination and refinement

Growth of FGFR1 crystals were grown by the hanging drop vapour diffusion method at 4 °C by mixing equal volumes of purified FGFR1 at 10 mg ml^−1^ with a reservoir solution comprising 18–20% PEG8000 (w/v), 200 mM ammonium sulphate, 100 mM PCTP, pH 6.75 and 20% ethylene glycol (v/v) so as to obtain a 2 μl drop^49^. Crystals were allowed to grow for at least 1 week before harvesting into a soaking solution comprising 22% PEG8000 (w/v), 200 mM ammonium sulphate, 100 mM PCTP, pH 6.75, 20% ethylene glycol (v/v) and 1 mM PDA or 1 mM dovitinib plus 1% DMSO (v/v). Soaks were incubated overnight. All work was carried out at 277 K. Crystals were flash frozen in a stream of nitrogen gas at 100 K directly from the drop. Diffraction data were collected in-house on a Rigaku FRE rotating anode generator (*λ*=1.54 Å) equipped with a Saturn 944 CCD detector or at Diamond Light Source on beamline I04 (*λ*=0.92 Å) using an ADSC Quantum 315 CCD detector. Data were processed with XDS and AIMLESS as implemented within autoPROC^51^ and XIA2 (ref. 52), respectively. The FGFR1–PDA and FGFR1–dovitinib crystals belong to the space group C1 2 1 and contain two complexes per asymmetric unit. The structures were solved by molecular replacement using the programme AMORE^53^ and an in-house FGFR1 structure as a search model. The structures were completed with iterative rounds of manual building in Coot^54^ interspersed with refinement using the programmes REFMAC^55^ and autoBUSTER applying NCS restraints and TLS. Quality checks were carried out using the validation tools in Coot and MolProbity^56^, while the compound stereochemistry was checked against the Cambridge Structure Database (CSD)^57^ using Mogul^58^. Ramachandran analysis revealed 93.6% (favoured), 6.0% (allowed) and 0.4% (generously allowed) for the FGFR1–PDA complex and 91.3% (favoured), 7.3% (allowed), 1.0% (generously allowed) and 0.4% (disallowed) for the FGFR1–dovitinib complex. Crystallographic statistics indicating data and model stereochemical quality are given in Supplementary Table 5. The final structures have been deposited in the PDB with ID code: FGFR1−PDA complex, 5A4C; FGFR1–dovitinib complex, 5A46. All structural figures were prepared using PyMOL (Schrödinger LLC).

### Surface plasmon resonance

Non-phosphorylated, histidine-tagged FGFR1 and non-phosphorylated, histidine-tagged Abl were immobilized as the ligand onto NTA sensor chips using a capture coupling method^59^. The NTA surface was first activated with 500 μM NiSO_4_ in immobilization buffer. The carboxymethyl dextran surface was then activated with a 1:1 ratio of 0.4 M EDC and 0.1 M NHS. Hexa-histidine-tagged protein was diluted into immobilization buffer to a concentration of 30 μg ml^−1^, and immobilized onto the surface with a 7-min injection. Remaining activated groups were blocked with 0.1 M Tris, pH 8.0. Typical immobilization levels ranged from 6,000 to 8,000 resonance units (RU). PBS, pH 7.4, 50 μM EDTA and 0.05% Surfactant P20 (v/v) (for Abl supplemented with 10% glycerol (v/v)) were used as immobilization buffer. Typical immobilization levels ranged from 3,800 to 8,000 RU. SPR experiments were performed using the Biacore 3000, Biacore S51 and Biacore T200 biosensors (GE Healthcare), with NTA and series S NTA sensor chips (GE Healthcare). All FGFR1 binding experiments were done using PBS (pH range 7.0–7.4), 50 μM EDTA, 0.05% Surfactant P20 (v/v) and 1% DMSO (v/v) or 50 mM Bis-Tris (pH range 5.5–6.5), 100 mM NaCl, 50 μM EDTA, 0.05% Surfactant P20 (v/v) and 1% DMSO (v/v), as running buffer. All Abl binding experiments were conducted using PBS, pH 7.4, 50 μM EDTA, 0.05% Surfactant P20 (v/v), 10% glycerol (v/v) and 1% DMSO (v/v) as running buffer. Compounds as DMSO stocks were diluted in DMSO to concentrations 100-fold higher than the final assay concentration. Finally, they were diluted 1:100 (v/v) in running buffer without DMSO to achieve the target concentration resulting in a final DMSO concentration of 1% (v/v).

### SPR kinetic analysis

To determine the rate constants of association (*k*_on_) or dissociation (*k*_off_), either multi-cycle or single-cycle SPR experiments were performed at 298 K. Single-cycle kinetic analysis was done at a constant flow rate of 60 μl min^−1^ in running buffer. The highest compound concentration varied, but for all analytes five sequential injections with constant injection time and a constant dilution factor were done. All analyte concentrations were injected in one cycle, one after the other for 120 s with a short dissociation phase in between injections (∼60 s) and with a longer dissociation phase at the end of the cycle (1,000 to 20,000 s that varied depending on the expected dissociation rate constant of the analyte). Zero-buffer blank injections were included for referencing. Biacore T200 evaluation software and BIAevaluation 4.1 software, respectively, were used for processing and analysing data. Rate constants were calculated globally from the obtained sensorgram data by fitting to a 1:1 interaction model. Representative sensorgrams are shown in Supplementary Fig. 6. Multi-cycle kinetic analysis was carried out as previously described^59^. Binding affinities (*K*_D_) were calculated from the equation *K*_D_=*k*_off_/*k*_on_.

### SPR thermodynamic analysis

The thermodynamic parameters of ponatinib and PDA binding were determined by performing single-cycle kinetic analysis at different temperatures as described above. SU5402 showed faster association and dissociation rate constants, thereby complicating kinetic analysis at higher temperatures. Binding affinities (*K*_D_) were therefore determined from dosage experiments and binding responses at equilibrium were fit to a 1:1 steady-state affinity model available within the Scrubber 2 software (BioLogic Software Ltd., Campbell, Australia). For each analysed ligand, rate constants and/or affinity were determined at a minimum of six different temperatures between 281 and 308 K. Association constants (*K*_A_) derived from kinetic or steady-state analysis were plotted as ln (*K*_A_) against 1/*T*, according to the integrated van't Hoff equation^60^,^61^—equation (1).




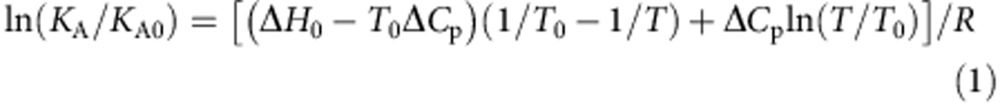




where *T*_0_ is an arbitrarily selected reference temperature, *K*_A0_ is the association constant at temperature *T*_0_, and Δ*H*_0_ is the van't Hoff enthalpy at temperature *T*_0_. Δ*C*_p_ is the temperature-independent heat capacity change (constrained to the experimentally determined values of −359 and −172 cal mol^−1 ^K^−1^ (Supplementary Fig. 7) for the analysis of PDA and SU5402, respectively) and *R*=1.986 cal mol^−1^ K^−1^. Δ*H*_0_ was determined by non-linear fitting of equation (1) to the experimental data using Prism 5.1 (GraphPad Software, Inc, La Jolla, USA). Transition state thermodynamic quantities were determined from the kinetic association (*k*_on_) and dissociation (*k*_off_) rate constants as previously described^64^ by plotting ln (*kh*/*k*_B_*T*) versus 1/*T* according to the linear Eyring equation (2).




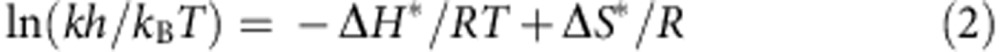




where *h*=6.63 × 10^−34^ J s and *k*_B_=1.38 × 10^−23^ J K^−1^ are the Planck and Boltzmann constants, respectively. Here *k* is either the association rate constant (*k*_on_) or the dissociation rate constant (*k*_off_). Δ*H* and Δ*S* are the changes in free enthalpy and entropy of binding, respectively, while the superscript ‘*' denotes that these refer to a transition state. *T* is the absolute temperature, and *R*=1.986 cal mol^−1^ K^−1^. ΔH* and ΔS* were determined by linear fitting of equation (2) to the experimental data using Prism 5.1 (Supplementary Fig. 4).

### Isothermal titration calorimetry

ITC experiments were carried out using an ITC_200_ instrument (Microcal Inc., GE Healthcare)^59^. Final ligand concentrations were achieved by diluting ligand stock solutions in DMSO 1:50 (v/v) in the experimental buffer, resulting in a final DMSO concentration of 2% (v/v). Protein concentration was determined by measuring the absorbance at 280 nm. DMSO concentration in the protein solution was adjusted to 2% (v/v). ITC measurements were routinely performed at 25 °C in 20 mM Tris, pH 7.8, 20 mM NaCl, 2 mM TCEP and 2% DMSO (v/v). The titrations were performed on 10−20 μM FGFR1 in the 200 μl sample cell using 2 μl injections of 0.1−0.2 mM ligand solution every 120 s. To correct for heats of dilution and mixing, the final baseline consisting of small peaks of identical size at the end of the experiment was subtracted. Representative ITC titrations are shown in Supplementary Fig. 2. To determine the heat capacity Δ*C*_p_ of ligands binding to FGFR1, ITC titrations were performed as described above at 10, 15, 20, 25, 30 and 35 °C. Binding enthalpies derived from ITC experiments were plotted as Δ*H* against *T* and Δ*C*_p_ are given by the slope of the linear regression analysis according to equation (3) (Supplementary Fig. 7):




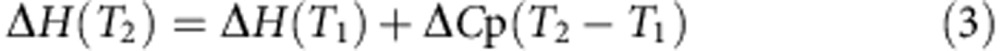




### Equilibrium chemical denaturation of FGFR1 using far-ultraviolet CD Spectroscopy

Far-ultraviolet CD spectra (190–260 nm) of FGFR1 kinase domain were obtained at different concentrations of GdmHCl. Spectra were measured on a JASCO J-810 spectropolarimeter at 293 K in 10 mM sodium phosphate buffer, pH 7.4; the concentration of FGFR1 used throughout was 2.8 μM. Unfolding experiments on FGFR1 kinase domain were completed by diluting the native FGFR1 protein sample with sequential additions of a second stock solution containing FGFR1 protein unfolded in 5 M GdmHCl, similarly buffered in 10 mM sodium phosphate, pH 7.4. The concentration of GdmHCl was determined using refractive index measurements as described^62^. Appropriate buffer blanks containing the corresponding concentration of denaturant were subtracted from all spectra, to account for the small contribution to the observed signal made by buffer. Molar ellipticity values at 222 nm obtained at varying denaturant concentrations were analysed using non-linear least-squares regression analysis, employing a modified version of the equation described in Morjana *et al*.^63^:




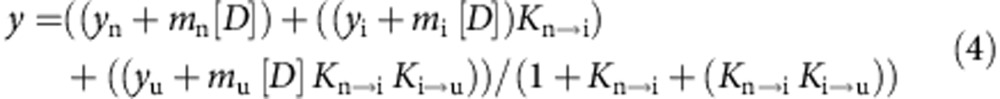




where *y*_n_, *y*_i_, *y*_u_ are the signals of the native (n), intermediate (i) and unfolded (u) states, respectively, at zero denaturant concentration ([*D*]), *m*_n_, *m*_i_, *m*_u_ represent d*y*/d[*D*] or slopes of the native, intermediate and unfolded state signals, respectively, *K*_n→i_=exp−(Δ*G*_n→i_−*m*_n→i_ [*D*])/*RT*, *K*_i→u_=exp−(Δ*G*_i→u_−*m*_i→u_ [*D*])/*RT*, where Δ*G* represents the free-energy change of the indicated transition, *m* is the slope of the free-energy change versus [*D*] for the indicated transition, *R* is the gas constant and *T* is the experimental temperature.

### NMR spectroscopy

Uniformly ^13^C/^15^N/^2^H-labelled samples of unbound FGFR1 and ligand-bound FGFR1–ponatinib and FGFR1–PDA complexes (1:1) were prepared^22^ as 0.35 mM solutions in 450 μl of 95% H_2_O and 5% D_2_O, 50 mM sodium phosphate, 0.1 mM EDTA, 2 mM dithiothreitol and 0.02% sodium azide (pH 7.0). PDA and ponatinib were added from concentrated stock solutions dissolved in DMSO-d6. NMR spectra were recorded at 298 K on Bruker Avance 600 MHz, Avance III 800 and Avance III HD 950 MHz spectrometers equipped with *z*-axis pulsed-field gradient TCI CryoProbes. TROSY-based detection schemes were used throughout as previously described^22^. Backbone resonance assignments for the FGFR1–PDA and FGFR1–ponatinib complexes followed standard triple-resonance strategies with two- and three-dimensional experiments using TROSY detection^22^, and will be reported elsewhere. The presence of backbone amide conformational exchange effects was studied by measuring the relaxation rates of the slowly relaxing ^15^N-{^1^H} TROSY doublet component using a Hahn-echo-based sequence optimized for deuterated proteins as described in Lakomek *et al*.^64^,^65^. All NMR data were processed using the NMRPipe suite of programmes^66^ and analysed with CARA^67^ to obtain assignments. ^15^N relaxation decay curves were fitted using a simplex search minimization and Monte Carlo estimation of errors.

### Hydrogen/deuterium-exchange mass spectrometry

Hydrogen exchange was performed using an HDX Manager (Waters Corp.) equipped with a CTC PAL sample handling robot (LEAP Technologies). Briefly, FGFR1 kinase domain (52.3 μM) in protonated aqueous buffer (20 mM Tris, 20 mM NaCl, 2 mM TCEP, pH 7.4) was incubated with ligand (100 μM) or DMSO. This gave 99.7 and 99.8% bound FGFR1 following dilution in the labelling solution for ponatinib (*K*_D_=7.7 nM) and PDA (*K*_D_=5.7 nM), respectively. Hydrogen exchange was initiated by dilution of 20-fold into deuterated buffer (20 mM Tris, 20 mM NaCl and 2 mM TCEP, pD 7.4) at 293 K. After incubation between 10 s and 2 h, hydrogen-exchange was quenched by mixing 1:1 with 100 mM potassium phosphate to a final pH of 2.55 at 274 K. Sample was immediately digested by a pepsin–agarose column (Poroszyme) and the resulting peptides separated on a C18 column (1 × 100 mm ACQUITY BEH 1.7 μm, Waters Corp.) with a linear gradient of acetonitrile (3–40%) supplemented with 0.1% formic acid. Peptides were analysed with a Synapt G2 mass spectrometer (Waters Corp.). Peptides were identified by MS^E^ fragmentation, yielding coverage of 97% of the His-tagged fusion protein construct of FGFR1 kinase domain with a high degree of redundancy (Supplementary Fig. 8). Peptides from Fig. 4c were confirmed by targeted tandem mass spectrometry fragmentation. No correction was made for back-exchange, and all results are reported as relative deuterium level. Deuterium incorporation was measured in DynamX (Waters Corp.) and data normalization was calculated with in-house software written in MatLab (Mathworks) and Python. Structural representations were generated with PyMol and plots in Fig. 4 prepared with Prism. Hydrogen/deuterium-exchange was represented in Fig. 4b by calculating the mean deuteration level per amino acid, according to equation (5).




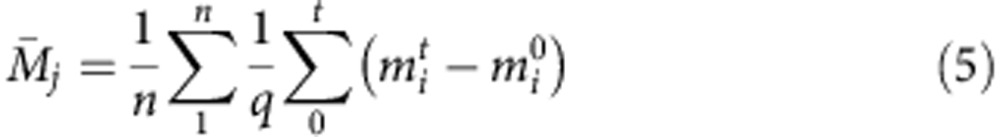




Where 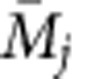 is the mean deuteration level at amino acid *j*, *n* is the number of overlapping peptides, *q* is the number of exchangeable amides for peptide species *i*, 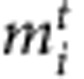 is the isotopic weighted midpoint at time *t* and 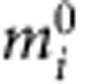 is the midpoint at time 0 (undeuterated).

## Additional information

**Accession codes:** coordinates and structure factors have been deposited in the Protein Data Bank with the following accession codes: FGFR1−PDA complex, 5A4C; FGFR1–dovitinib complex, 5A46.

**How to cite this article:** Klein, T. *et al*. Structural and dynamic insights into the energetics of activation loop rearrangement in FGFR1 kinase. *Nat. Commun.* 6:7877 doi: 10.1038/ncomms8877 (2015).

## Supplementary Material

Supplementary Information Supplementary Figures 1-9 and Supplementary Tables 1-5

## Figures and Tables

**Table 1:** Standard enthalpies, entropies and Gibbs free energies (kcal mol^−1^) for binding of the type I inhibitors, PDA and SU5402, and the type II inhibitor ponatinib, derived from non-linear van't Hoff analysis of data in Fig. 2b.

Inhibitor	Δ*H*_0_^van't Hoff^	−*T*Δ*S*_0_^van't Hoff^*	Δ*G*_0_^van't Hoff^*	*R* ^2^ †
PDA	−11.5±0.8	0.4±0.8	−11.1±0.02	0.862
SU5402	−14.2±0.8	4.8±0.8	−9.5±0.02	0.983
Ponatinib	10.1±1.6	−21.0±1.6	−10.9±0.04	0.879

^*^Standard errors, *T*_0_=298 K.

^†^From non-linear fitting of data in Fig. 3b.
