# Supplementary material for: Structural and dynamic insights into the energetics of activation loop rearrangement in FGFR1 kinase
Source: Nat Commun. 2015 Jul 23;6:7877. doi: 10.1038/ncomms8877 (PMC4525181; doi:10.1038/ncomms8877)
Supplement: Supplementary Information — Supplementary Figures 1-9 and Supplementary Tables 1-5 [file ncomms8877-s1.pdf]

Supplementary Figures

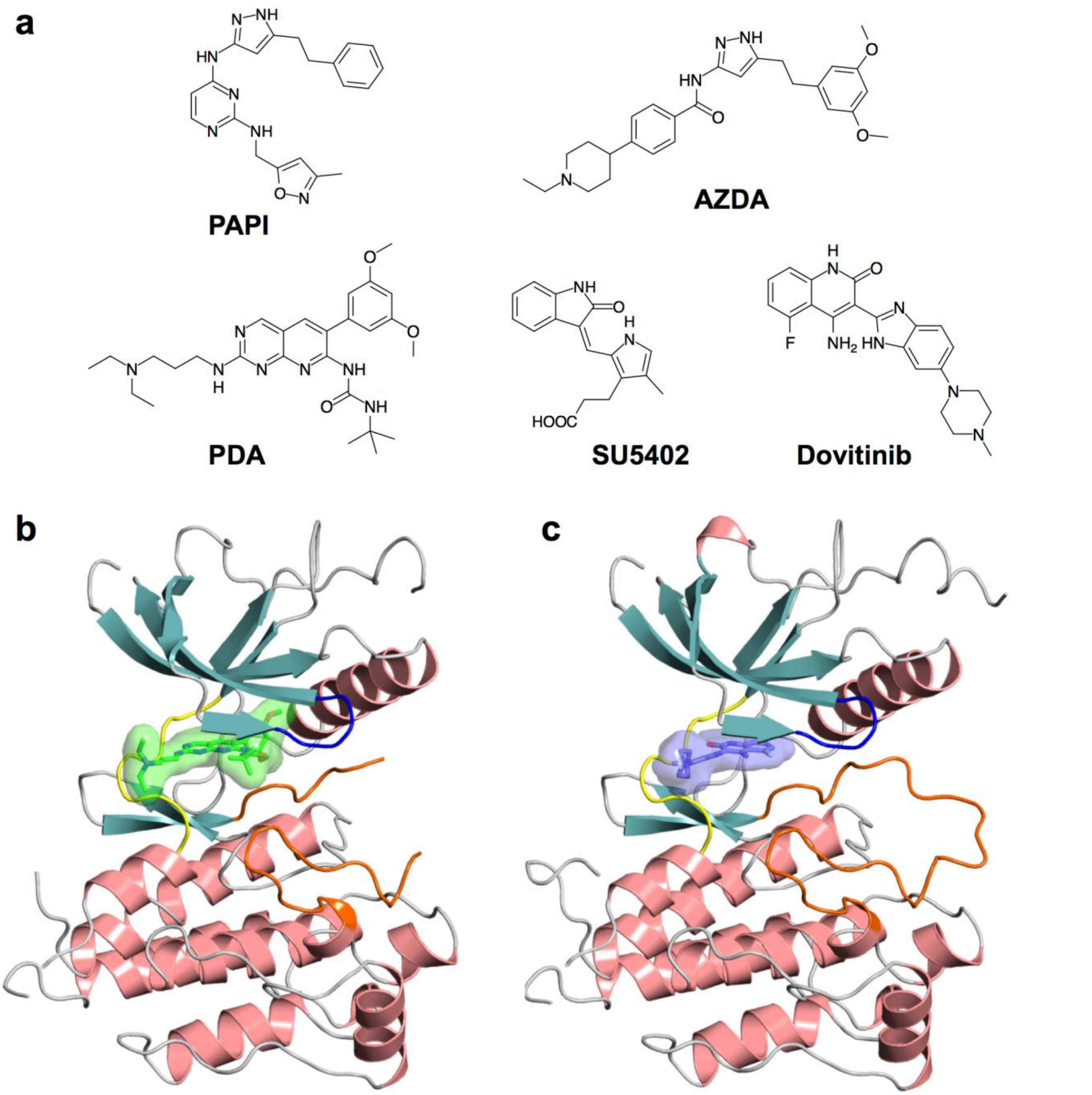

Supplementary Figure 1 Type I FGFR1 inhibitors (**a**) Chemical structures of a pyrazolylaminopyrimidine inhibitor (henceforth referred to as PAPI; PDB-code of the FGFR1-PAPI complex: 4NKS), a close analog of AZD4547 (henceforth referred to as AZDA; PDB-code of the FGFR1-AZD4547 complex: 4V05), a close analog of PD 173074 (henceforth referred to as PDA), the indolinone SU5402 (PDB-code of the FGFR1-SU5402 complex: 1FGI), and the 4-amino-3-benzimidazol-2-ylhydroquinolin-2-one dovitinib. (**b**) Ribbon representation of the X-ray crystal structure of FGFR1 in complex with PDA (green). The hinge region (yellow), P-loop (blue), and activation loop (orange) are highlighted. (**c**) Ribbon representation of the X-ray crystal structure of FGFR1 in complex with dovitinib (slate blue). Protein chain colored as in (b).

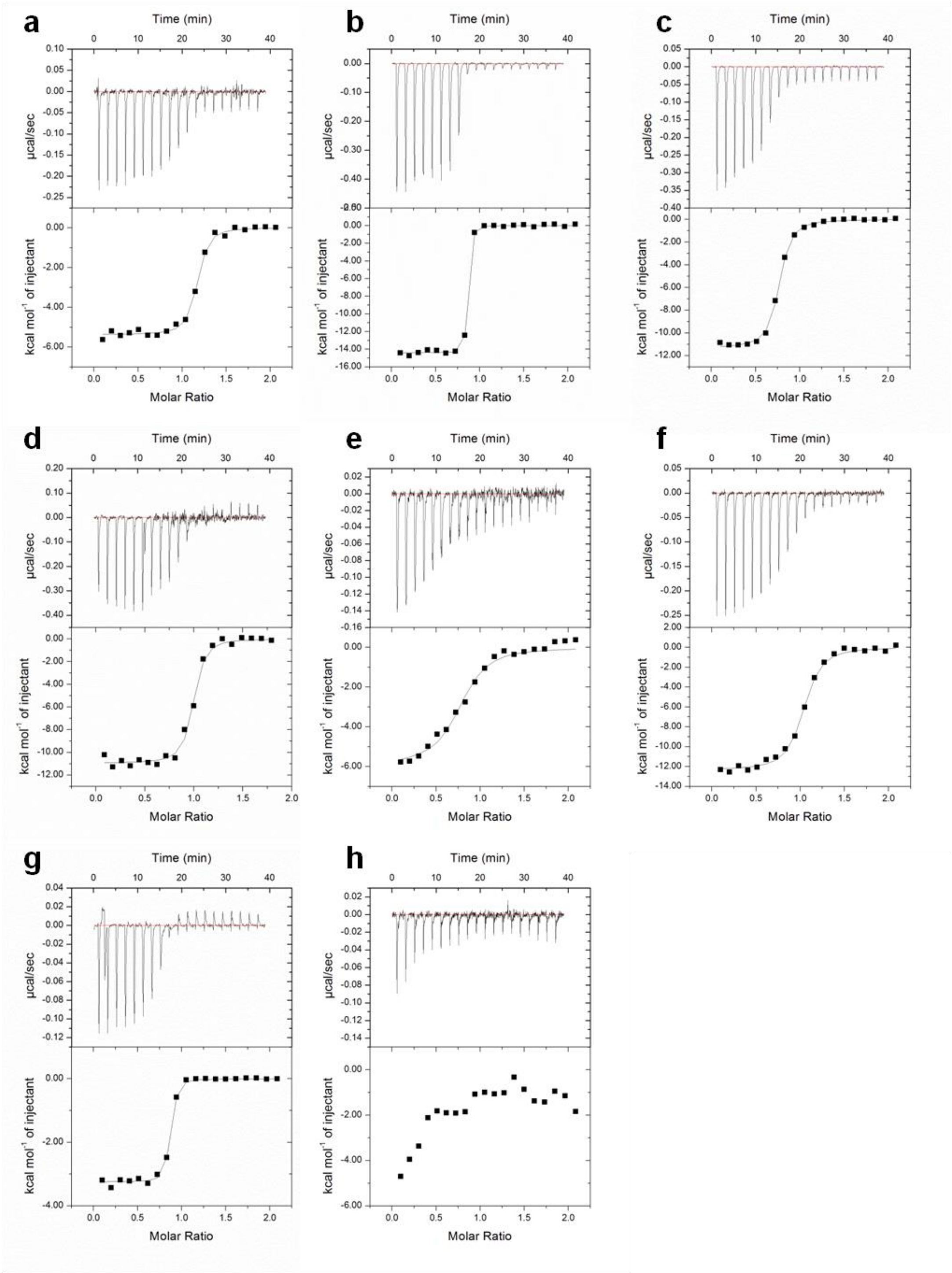

Supplementary Figure 2 Representative ITC titrations. (**a**) PAPI (0.2 mM) against FGFR1 (20 µM). (**b**) AZDA (0.2 mM) against FGFR1 (200 µM). (**c**) **2** (0.2mM) against FGFR1 (20 µM) in the presence of 40 µM **1**. (**d**) PDA (0.2 mM) against FGFR1 (20 µM). (**e**) PDA (0.2mM) against FGFR1 (20 µM) in the presence of 32 µM PAPI. (**f**) SU5402 (0.1 mM) against FGFR1 (10 µM). (**g**) Dovitinib (0.2 mM) against FGFR1 (20 µM). (**h**) ITC titration of Ponatinib (0.1 mM) against FGFR1 (10 µM). The recorded change in heat is shown in units of µcal sec^{-1} as a function of time for successive injections of the ligand (upper panels). Integrated heats (black squares) plotted against the molar ratio of the binding reaction. The continuous line represents the results of the non-linear least squares fitting of the data to a binding model (lower panels).

a

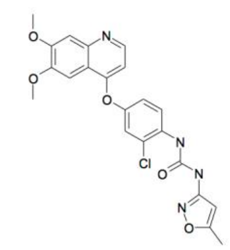

b

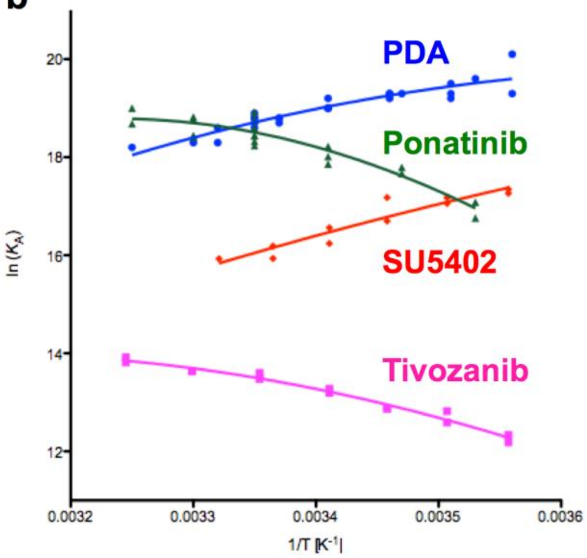

c

PDA (R^2 = 0.862)

ΔH_0 van't Hoff = -11.491 ± 0.773 kcal mol^{-1}

ΔG_0 van't Hoff = -11.093 ± 0.023 kcal mol^{-1}

-$T\Delta S_0$ van't Hoff =0.398 ± 0.773 kcal mol^{-1}

SU5402 (R^2 = 0.897)

ΔH_0 van't Hoff = -14.208 ± 1.457 kcal mol^{-1}

ΔG_0 van't Hoff = -9.520 ± 0.052 kcal mol^{-1}

-$T\Delta S_0$ van't Hoff =4.688 ± 1.458 kcal mol^{-1}

Ponatinib (R^2 = 0.879)

ΔH_0 van't Hoff = 10.088 ± 1.563 kcal mol^{-1}

ΔG_0 van't Hoff = -10.947 ± 0.035 kcal mol^{-1}

-$T\Delta S_0$ van't Hoff =-21.035 ± 1.563 kcal mol^{-1}

Tivozanib (R^2 = 0.972)

ΔH_0 van't Hoff = 7.787 ± 1.352 kcal mol^{-1}

ΔG_0 van't Hoff = -7.984 ± 0.022 kcal mol^{-1}

-$T\Delta S_0$ van't Hoff = -15.771 ± 1.352 kcal mol^{-1}

Supplementary Figure 3 (**a**) Structure of tivozanib. (**b**) Van't Hoff plot visualization of temperature-dependent FGFR1-ligand interactions measured by SPR for type I inhibitors PDA (blue circles) and SU5402 (red diamonds); and type II inhibitors ponatinib (green triangles) and tivozanib (K_D = 1.3 μM; magenta squares). (**c**) Thermodynamic parameters derived from non-linear least-squares fitting of the temeprature-dependent inhibitor binding data in (b).

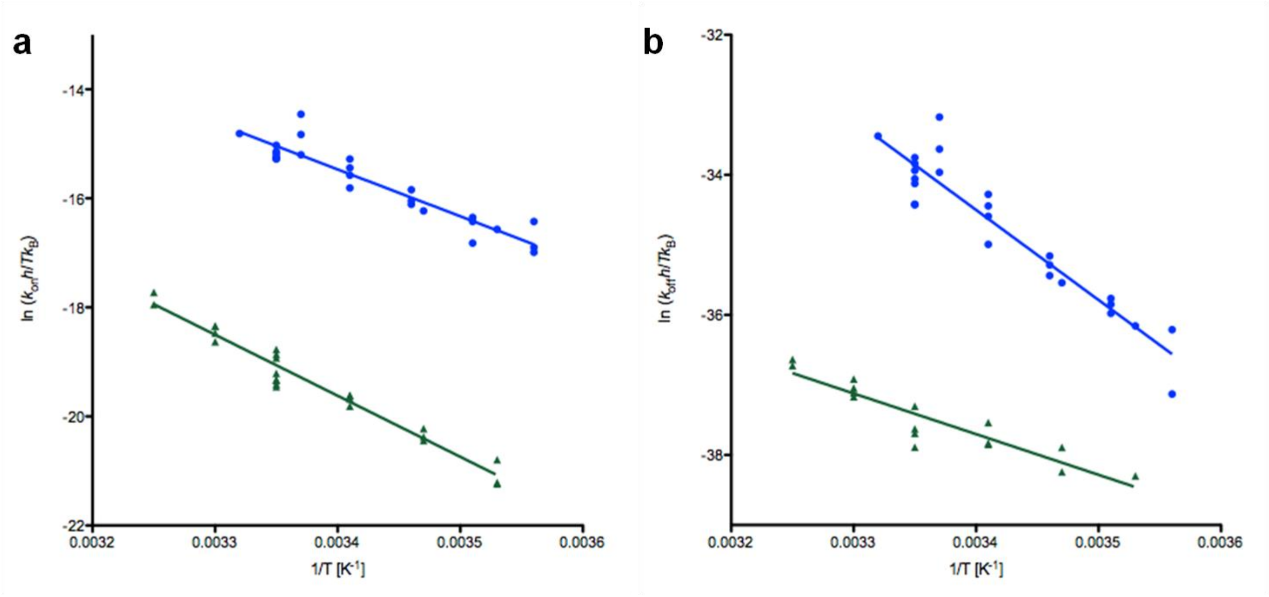

Supplementary Figure 4 Transition state free energies. Linear Eyring plots for PDA (blue circles) and ponatinib (green triangles) plotted using either k_{on} (**a**) or k_{off} (**b**) illustrate the temperature dependence of the PDA/FGFR1 and ponatinib/FGFR1 kinetics, where $-\Delta H^{\#}/R$ is given by the slope and $\Delta S^{\#}$ is given by the y intercept. Linear regressions were performed using GraphPad Prism v5.1 which yielded correlation coefficients of 0.879 (PDA) and 0.958 (ponatinib) for the k_{on} data and 0.891 (PDA) and 0.845 (ponatinib) for the k_{off} data.

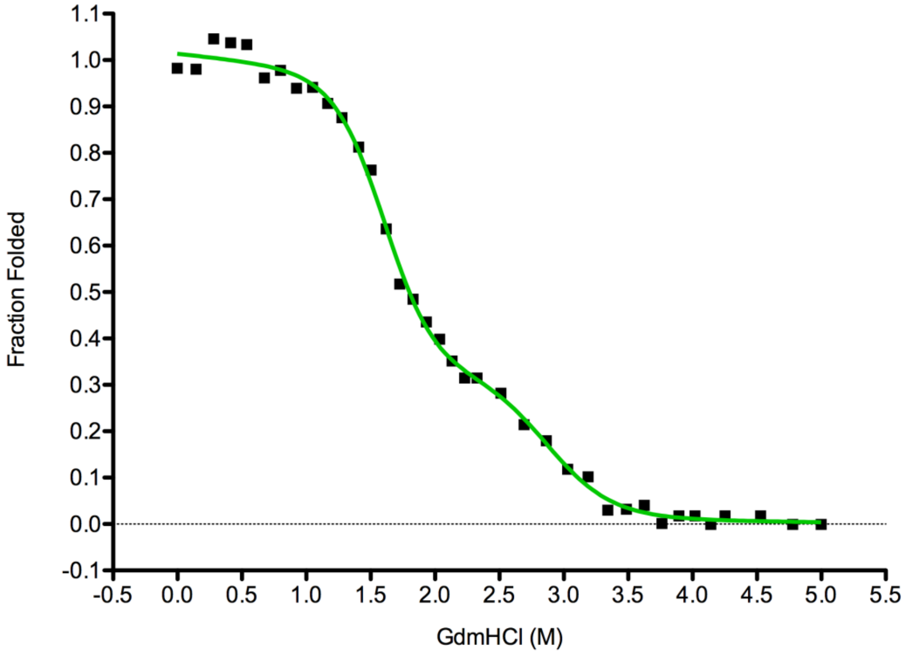

Supplementary Figure 5 GdmCl-induced unfolding transition curve of FGFR1 monitored by the change in far-UV CD. The fraction of folded protein is calculated from the change in molar ellipticity at 222 nm and plotted as a function of GdmCl concentration. Data were fitted to an equation describing a three-state transition (green line, R^2= 0.998).

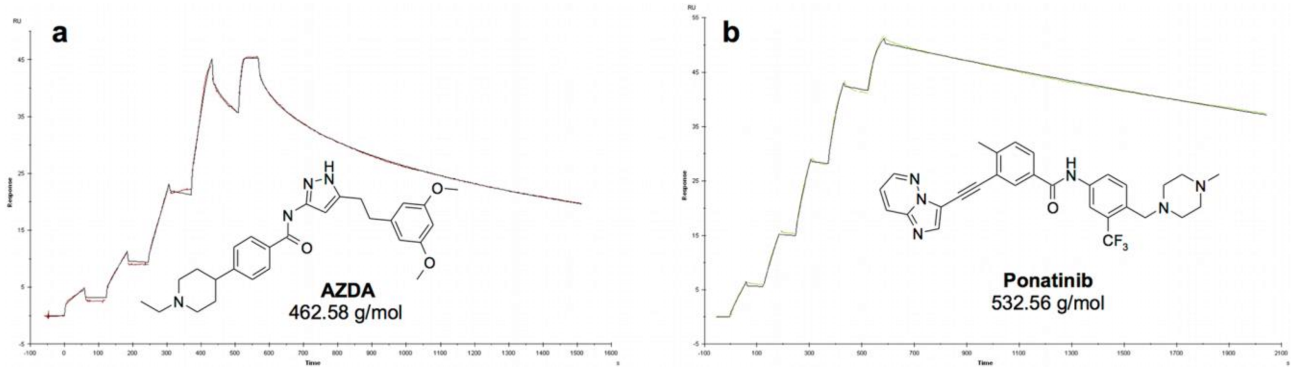

Supplementary Figure 6 Representative sensorgrams for single-cycle kinetic analysis of inhibitors interacting with FGFR1 immobilised to similar densities (3800 - 5711 RU). (**a**) AZDA at concentrations of 3.9, 7.8, 15.6, 31.3, and 62.5 nM was injected in a single cycle one concentration after the other. (**b**) Ponatinib at concentrations of 78.1, 156.3, 312.5, 625.0, and 1250.0 nM was injected in a single cycle one concentration after the other. Data were globally fit to a 1:1 interaction model as shown by the black lines. The compound structure, name, and molecular mass are provided on each data set.

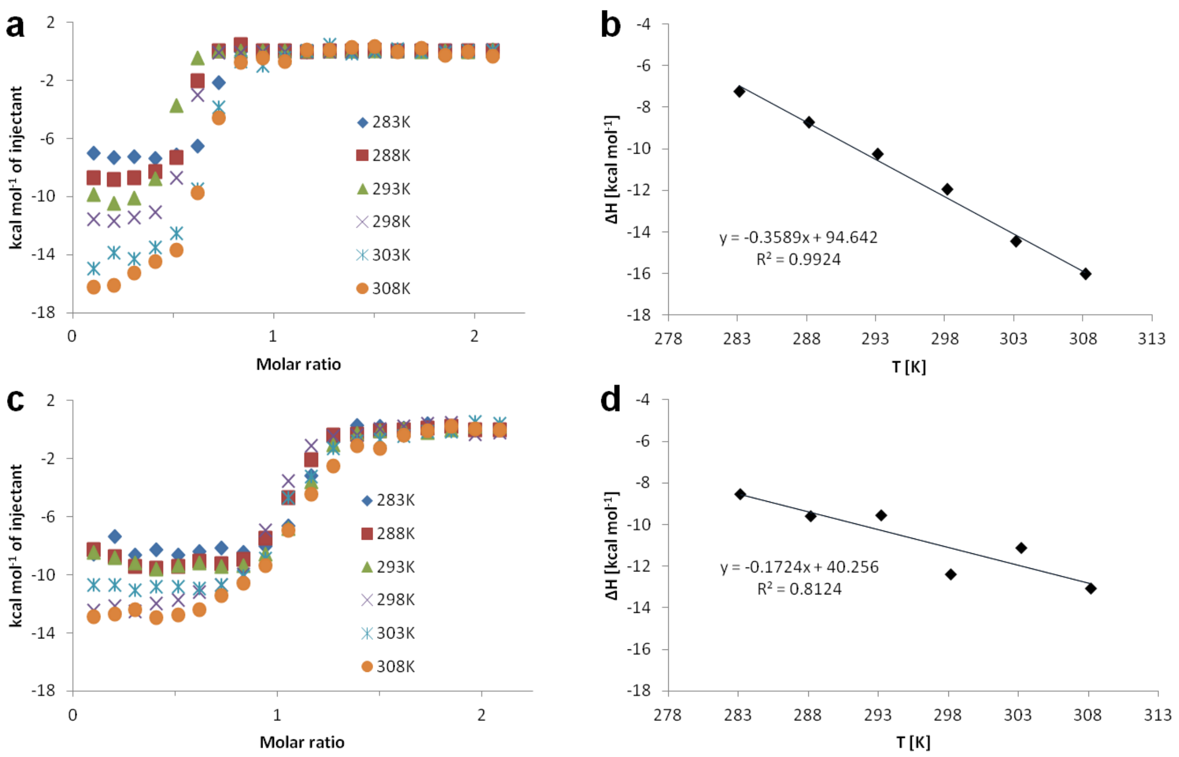

Supplementary Figure 7 Determination of heat capacity ΔC_p. (**a**) Integrated heats plotted against the molar ratio of the binding reaction for ITC titrations of PDA (200 µM) against FGFR1 (20 µM) at different temperatures. (**b**) Correlation of binding enthalpy Δ*H* with temperature for PDA. (**c**) Integrated heats plotted against the molar ratio of the binding reaction for ITC titrations of SU5402 (100 µM) against FGFR1 (10 µM) at different temperatures. (**d**) Correlation of binding enthalpy Δ*H* with temperature for SU5402.

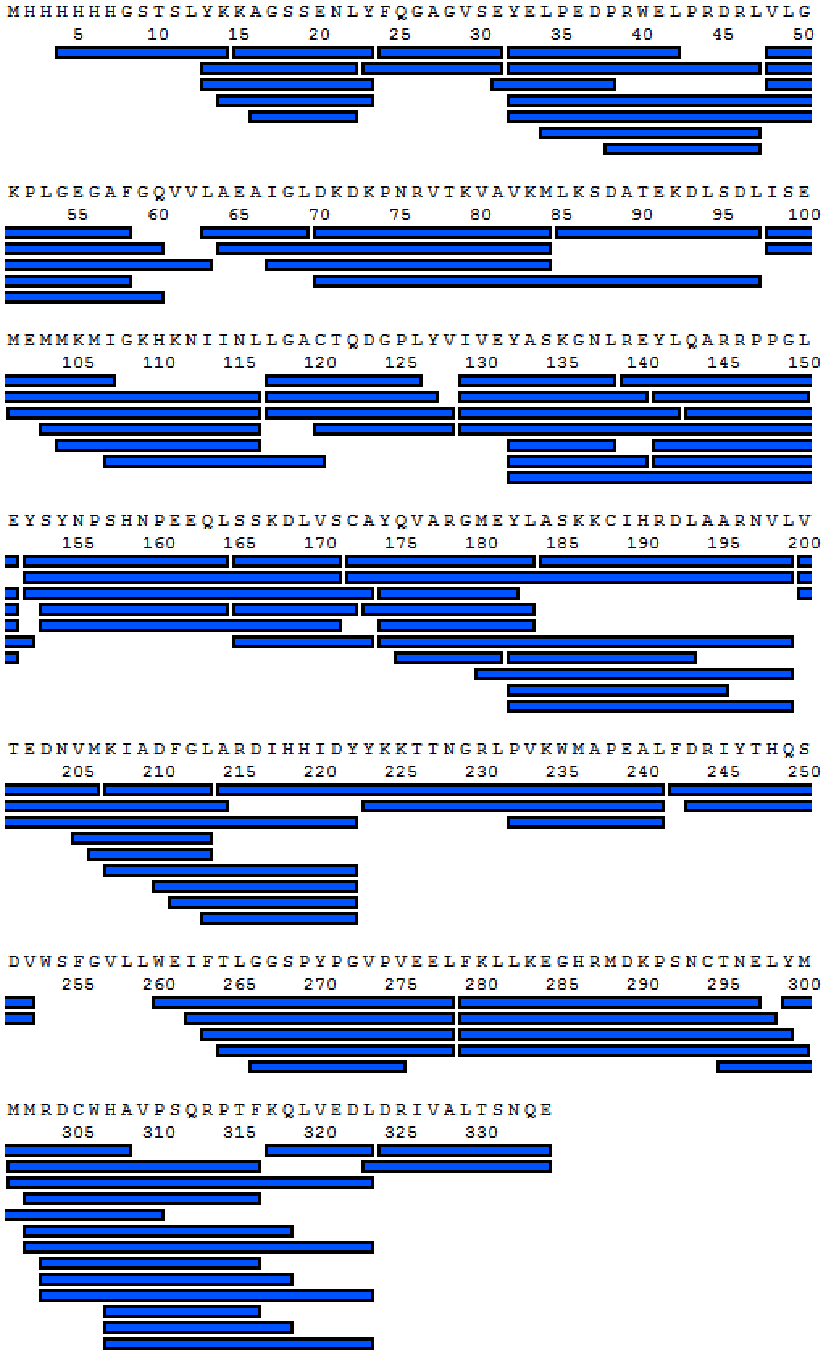

Total: 97.0% Coverage, 4.63 Redundancy

Supplementary Figure 8 Coverage map for hydrogen/deuterium-exchange mass spectrometry (HDX-MS) of FGFR1 kinase domain. Total coverage of the tagged fusion protein was 97.0 %, with good redundancy (average 4.6 unique peptides reporting on each amino acid) that serves to increase confidence and net resolution of the HDX data. Each blue bar denotes a peptide resulting from pepsin digestion of FGFR1 under the conditions used for HDX-MS. HDX-MS data for these peptides is represented in Fig. 4. Arbitrary numbering is displayed here.

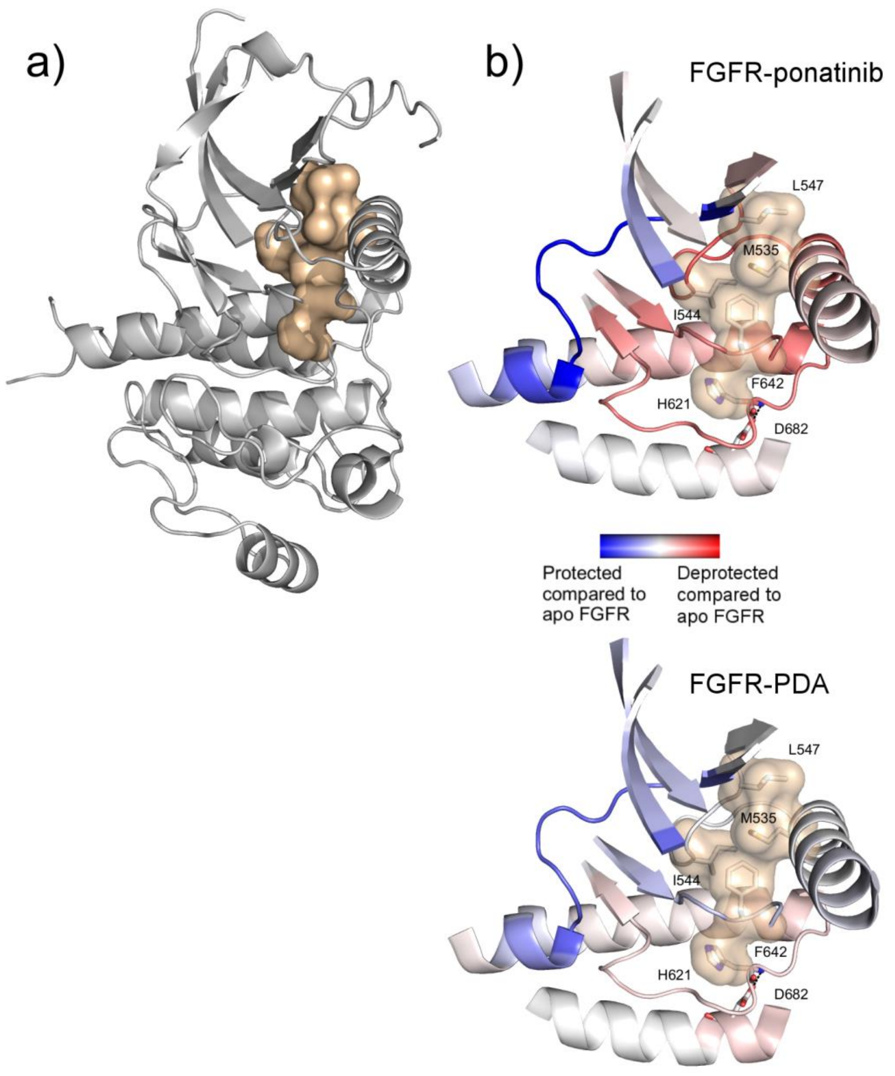

Supplementary Figure 9 Regulatory (R) spine solvent exposure in response to binding of ponatinib or PDA. (**a**) Kinase domain with the five residues that comprise the R spine shown as a continuous surface (Met535, Ile544, Leu547, His621 and Phe642). (**b**) Detail of the R spine. Coloring is for the HDX-MS data in complex with ponatinib (top) or PDA (bottom) versus free FGFR1. R spine amino acids are shown as sticks with elemental coloring, also showing Asp682 from the αF helix that makes a hydrogen bond (dashed line) with His621.

Supplementary Tables

Supplementary Table 1 Kinetic data for selected type I and type II inhibitors binding to FGFR1 kinase domain*,†

cmpd	k_{on} [M^{-1} s^{-1}]	k_{off} [s^{-1}]	K_D [nM]
**PAPI**	$3.8x10^6$±$7.7x10^4$	$2.5x10^{-1}$±$7.1x10^{-3}$	67.9±2.2
**AZDA**	$2.9x10^7$±$6.3x10^5$	$2.1x10^{-2}$±$4.8x10^{-4}$	0.7±0.02
**PDA**	$1.6x10^6$±$5.8x10^3$	$9.2x10^{-3}$±$3.3x10^{-5}$	5.7±0.03
**SU5402**	$1.5x10^6$±$1.7x10^4$	$1.1x10^{-1}$±$7.7x10^{-4}$	70.9±0.9
**Dovitinib**	$2.5x10^6$±5.4 $x10^4$	$5.5x10^{-2}$±1.3 $x10^{-3}$	22.1±0.3
**Ponatinib**	$2.4x10^4$±$7.2x10^2$	1.9 $x10^{-4}$±$1.8x10^{-5}$	7.9±0.8

*SPR experiments were performed at 298.15 K and pH=7.4. †Equilibrium dissociation constants (k_{on}/k_{off}). Data represent mean ± SE from at least three independent experiments.

Supplementary Table 2 Thermodynamic data for selected type I inhibitors binding to FGFR1 kinase domain*

cmpd	N	ΔH [kcal mol^{-1}]	$-T\Delta S$ [kcal mol^{-1}]	ΔG [kcal mol^{-1}]	K_D [nM]	n	ΔC_p [cal mol^{-1} K^{-1}]
**PAPI**	1.1±0.01	-5.7±0.4	-4.0±0.4	-9.8±0.1	69.8±9.6	2	-
**AZDA**	0.8±0.001	-15.0±0.6	3.0±0.4	-12.0±0.1	1.6±0.3	2	-
**PDA**	0.8±0.02	-12.1±1.8	1.1±0.0	-11.0±0.0	8.8±0.0	2	-359
**SU5402**	1.0±0.04	-12.4±0.2	2.7±0.2	-9.7±0.1	76.1±13.0	3	-172
**Dovitinib**	0.7±0.01	-3.7±0.4	-6.7±0.5	-10.4±0.1	24.9±4.5	2	-

*ITC titrations were performed at 298.15 K. Data represent mean ± SD.

Supplementary Table 3 Transition state free energies determined from linear Erying plots for selected inhibitors binding to FGFR1 kinase domain

cmpd	$\Delta H^{\#}_{ass}$ [kcal mol^{-1}]	$-T\Delta S^{\#}_{ass}$ [kcal mol^{-1}]	$\Delta G^{\#}_{ass}$ [kcal mol^{-1}]	$\Delta H^{\#}_{diss}$ [kcal mol^{-1}]	$-T\Delta S^{\#}_{diss}$ [kcal mol^{-1}]	$\Delta G^{\#}_{diss}$ [kcal mol^{-1}]
**PDA**	16.6±1.3	-7.9±1.3	8.7±1.8	24.8±1.8	-5.3±1.8	19.5±2.6
**Ponatinib**	22.2±1.1	-11.2±1.1	11.0±1.6	10.9±1.4	10.6±1.4	21.5±2.3

$\Delta H^{\#}_{ass}$, $-T\Delta S^{\#}_{ass}$, and $\Delta G^{\#}_{ass}$ are transition state enthalpy, entropy, and free binding energy, respectively, for the association phase; $\Delta H^{\#}_{diss}$, $-T\Delta S^{\#}_{diss}$, and $\Delta G^{\#}_{diss}$ are transition state enthalpy, entropy, and free binding energy, respectively, for the dissociation phase. Errors indicate SEM from at least three independent experiments.

Supplementary Table 4 Kinetic data for ponatinib and imatinib binding to ABL kinase domain

	ABL		
**cmpd**	k_{on} [M^{-1} s^{-1}]	k_{off} [s^{-1}]	K_D [nM]
Ponatinib	$5.2x10^5$±$1.8x10^5$	3.6 $x10^{-4}$±$1.3x10^{-4}$	0.9±0.4
Imatinib	$5.5x10^5$±$2.3x10^4$	8.4 $x10^{-4}$±$3.2x10^{-6}$	1.5±0.1

[a]SPR experiments were performed at 298.15 K. Data represent mean ± SE from at least three independent experiments.

Supplementary Table 5 Data collection and refinement statistics (molecular replacement)

	FGFR1-PDA complex	FGFR1-Dovitinib complex
**Data collection**		
Space group	C 1 2 1	C 1 2 1
Cell dimensions		
a, *b*, *c* (Å)	208.43, 57.46, 65.67	209.80, 56.82, 65.74
α, β, γ (°)	90, 107.34, 90	90, 107.36, 90
Resolution (Å)	99.48-2.09 (2.25-2.09)[a]	62.75-2.63 (2.94-2.63)
R_{merge}	0.041 (0.401)	0.068 (0.487)
$I / \sigma I$	20.1 (2.6)	12.3 (2.8)
Completeness (%)	97 (86.7)	97.1 (98.9)
Redundancy	3.4 (2.4)	3.4 (3.5)
**Refinement**		
Resolution (Å)	28.73-2.09 (2.14-2.09)	62.75-2.63 (2.76-2.63)
No. reflections	42861 (2334)	21565 (2732)
R_{work} / R_{free} (%)	20.1/23.1 (24.6/25.3)	17.8/24.6 (20.8/28.5)
No. atoms		
Protein	4499	4461
Ligand/ion	74	58
Water	348	228
B-factors ($Å^2$)		
Protein	50.9	83.5
Ligand/ion	43.7	86.2
Water	55.4	61.2
R.m.s. deviations		
Bond lengths (Å)	0.010	0.010
Bond angles (°)	1.06	1.14

[a]Values in parentheses are for the highest-resolution shell. All data were collected from one crystal.
